# Story contents and intensity of the anxious symptomatology in children and adolescents with Autism Spectrum Disorder

**DOI:** 10.1080/02673843.2020.1737156

**Published:** 2020-03-17

**Authors:** Giuseppe Iandolo, Laura López-Florit, Paola Venuti, Michelle J.Y. Neoh, Marc H. Bornstein, Gianluca Esposito

**Affiliations:** a Departamento de Psicología, Facultad de Ciencias Biomédicas, Universidad Europea de Madrid, Madrid, Spain;; b Department of Psychology and Cognitive Science, University of Trento, Rovereto, Italy;; c Psychology Program, School of Social Sciences, Nanyang Technological University, Singapore, Singapore;; d Child and Family Research, Eunice Kennedy Shriver National Institute of Child Health and Human Development, Bethesda, MD, USA;; e Institute for Fiscal Studies, London, UK;; f Lee Kong Chian School of Medicine, Nanyang Technological University, Singapore

**Keywords:** Storytelling, narrative cohesion, narrative development, Autism Spectrum Disorder, anxiety

## Abstract

This study aimed to analyse and compare the storytelling of 25 children with Autism Spectrum Disorder (ASD) with a comparison group of 25 children with typical development. Children’s narratives were transcribed verbatim, and their forms and contents were analysed. The two groups were matched according to the narrative cohesion of the story using the Bears Family Projective Test, equivalent verbal age, sex, and socioeconomic level. No differences in the forms of the stories emerged, but compared with the narratives of the typical development group, the narrative contents of the ASD group showed more adaptive and maladaptive behaviours of the characters, more problems, and less use of the atmosphere outside the home. These contents are related to the intensity of the anxious symptomatology indicated by the ASD group, their family members and teachers.

## Introduction

Narration is an avenue for thought, communication and sharing reality is an important sociocultural activity through which human beings organize and integrate experiences in order to better understand them (Bamberg & Andrews, 2004; Bamberg, 2012; Bruner, 1991; Ochs & Capps, 2001). Conversational narratives are a major way that children share experiences, which supports the development of emotional attachment and social relationships (Von Klitzing et al., 2007). The quality of the construction of one’s narrative may influence the assimilation of new experiences into their sense of self. Moreover, the creation of a narrative regarding a conflict or stressful event is fundamental to its incorporation into one’s self-representation (Van der Kolk & Fisler, 1995).

Storytelling requires the coordination of a number of different linguistic, social and cognitive abilities including emotional and social skills as well as knowledge and memory of the structure of events, cohesion of the narrative, executive planning and theory of mind (Flynn, 2018; Holmes et al., 2019; Leslie et al., 2006). The global organization of a child’s cognitive, affective and narrative functioning includes factors such as their temperamental dispositions, emotional experiences, social learning that associates primary emotions with more complex secondary emotions (Bartlett & Bartlett, 1932; Botvin & Sutton-Smith, 1977; Nelson, 1989). From this perspective, the representations that converge in a narrative are formed by meanings and situations extracted from experiences and integrated into the memory (Iandolo et al., 2012a), to which positive, negative emotional and relational aspects are associated.

In recent decades, there has been a significant increase in research on narration and narrative competence in the context of Autism Spectrum Disorders (ASD). In the Diagnostic and Statistical Manual of Mental Disorders 5 (DSM-5; American Psychiatric Association [APA], 2013), ASD is a neurodevelopmental disorder characterized by impairments in social communication and interaction, repetitive behaviours and restricted interests. ASDs are characterized by their heterogeneity, multiple and complex aetiologies, and variability in levels of affect and associated symptoms (Artigas & Paula, 2016). In addition to these core features, ASD is usually comorbid with at least one mental health condition, with anxiety being one of the most common with Significant anxiety has been reported in around 50% of children and adolescents (Simonoff et al., 2008; Van Steensel et al., 2011). Studies have also looked into the relationship between emotion dysregulation (Geller, 2005) and emotional difficulties and maladaptive emotional responses such as irritability, poor anger control and mood dysregulation (Lecavalier et al., 2006; Prizant & Laurent, 2011) in individuals with ASD. Emotional difficulties have also been shown to manifest in one’s narratives. In a previous study by Iandolo et al. (2012a) has also found that stories of children with emotional difficulties showed many unsolved problematic events, unclear characters and negative relationships and behaviours. Meyer et al. (2006) investigated the possibility that emotional and behavioural difficulties in children with ASD were associated with alterations in information processing and social attribution. In their results, children with ASD showed poor psychosocial adaptation, scarce social information and poor patterns of attributional processing, leading Meyer to surmise that cognitive and social-cognitive skills in ASDs were associated with a bias in information processing, but not directly with emotional and behavioural difficulties. Hence, the study suggested that behavioural symptoms and comorbid anxiety in ASD could be associated with the perceptual style, understanding and social experience of people with ASD.

Although language abilities of individuals with ASD are considerably heterogenous, language and communication difficulties, including impairments in narration, are observed across the ASD spectrum (Tager-Flusberg, 2005). The communicative profile of individuals with ASD is characterized by an alteration in the pragmatics of language, based on social-cognitive skills, which contrasts with an adequate explanation of the formal structure (Bartolucci et al., 1980). Research on narration in ASD children have found that the narratives of ASD children tend to be shorter (Siller et al., 2014), less grammatically complex and less coherent (Loveland & Tunali, 1993). Other characteristics of the narratives of ASD children include (a) difficulties adopting an externalized point of view and introducing personal experiences and causal explanations; (b) a lower propensity for explanations about the mental states of the characters; (c) less use of the memory of a story to organize a coherent and cohesive narrative around a central theme (Diehl et al., 2006); (d) lesser use of personal pronouns, temporal and referential expressions. Their stories also contain fewer evaluative measures such as the use of internal state language and causal statements (Capps et al., 2000; King et al., 2013; Siller et al., 2014). Overall, a meta-analysis of the narrative performance of high-functioning ASD individuals found that individuals with ASD showed deficits in the following narrative domains (i) *microstructure* – internal linguistic structures including productivity and grammar, (ii) *macrostructure* – overall content and hierarchical organization, and (iii) *internal state language* – vocabulary describing characters’ perceptions, emotions and thoughts (Baixauli et al., 2016).

The cognitive systems involved in the triad of behavioural impairments in ASD (see Happé et al., 2006; Happé & Ronald, 2008) have been proposed to be related to the observed impairments in narration in individuals with ASD. Firstly, individuals with ASD may present a deficit in theory of mind (ToM) with difficulties in the representation of the inner world of others and socially in general (Baron-Cohen et al., 1986) and the ability to empathize. The understanding of one’s own and another’s mind is based on the understanding of the psychological and emotional states, and allows the narration of one’s own experiences (Guidano, 1987) and development of personal episodic memory (Solcoff, 2001, 2002). Hence, it has been suggested that these ToM deficits would result in individuals with ASD having difficulties with identifying psychological states in characters of the story and adapting the narrative according to shared knowledge with the audience; of which an association between the narrative performance of children with ASD and their performance on ToM tasks has been confirmed (Tager-Flusberg & Sullivan, 1995). Secondly, cognitive alterations in ASD have been proposed to be caused by difficulties with executive functioning – processes including planning, working memory, inhibition control and flexibility. According to the Weak Coherence Account (Happé & Frith, 2006), individuals with ASD have a natural tendency to focus on local information and show difficulty in incorporating these local features into meaningful representations. Another aspect of the Weak Coherence Account – utilizing context in sense making – has been proposed to be lacking in this contextual sensitivity, thereby impeding the use of context in sense making. Thirdly, difficulties with episodic future thinking and mental time travel have recently been associated with narrative patterns of individuals with ASD. Mental time travel, the skill that allows humans to navigate in time (Suddendorf & Corballis, 1997, 2007), has been argued to be involved in the processing of a narrative and generation of a globally coherent narrative. Given the impairments in episodic memory and episodic future thinking in individuals with ASD, individuals with ASD may have difficulty with mentally projecting in time. In a study by Ferretti et al. (2018) analysing the relation between mental time travel and producing a globally coherent narrative, a subgroup of children with ASD had impaired episodic future thinking skills and performed significantly worse on the narration production task.

Hence, the aim of the present study was to (i) analyse and compare the stories of the two groups (ASD vs. TD) in terms of narrative structure and content, and (ii) investigate the relationship of the narrative structure and content of these stories to the age and behaviour in different contexts (home, school and self-perception) of the children.

## Method

### Design

The design of the study is cross-sectional and compared the narratives in the Bears Family Projective Test (Bornstein & Tamis-lemonda, 1995; Iandolo, 2011) of 25 children with Autism Spectrum Disorder level 1 (ASD) and 25 children with typical development (TD). Four hypotheses were explored:

Hypothesis 1: The structure of the story (cohesion and narrative structure) improves with age in both groups as it relates to the linguistic maturity and vocabulary of the participants.

Hypothesis 2: At the same level of narrative cohesion of the story, the number of propositions and episodes present in the story of the ASD group is higher than the TD group.

Hypothesis 3: There are differences in story content between the ASD group and TD group. Stories of the ASD group will show more adaptive and maladaptive behaviours of the characters, more problems, and less use of the external environment outside the home as the story’s setting.

Hypothesis 4: The different story contents present in the ASD group may be related to greater exposure to experiences and representations of relational and behavioural difficulties in different contexts.

### Participants

The study involved 50 Spanish boys between 5 and 18 years of age: 25 participants in the ASD group and 25 participants in the TD group. The participants of the ASD group were selected from users of a Spanish psychological centre who had the verbal skills to perform a narrative task. All the children in the ASD group had received a previous diagnosis of Autism Spectrum Disorder based on the DSM-5 (APA, 2013) through the ADOS or ADOS-2 (Lord et al., 2015). All the participants in this study were males in accordance with the literature on ASD prevalence indicating that males are diagnosed four times more than girls (see Morales-Hidalgo et al., 2018) and the male bias in ASD prevalence where boys are over-represented among high-functioning cases (Banach et al., 2009; Werling & Geschwind, 2013). The TD group was part of a previous study with Spanish children and adolescents with typical development and without emotional or behavioural difficulties (Iandolo, 2011). The two groups were matched according to sex, socioeconomic level, the equivalent verbal age based on IQ – some adolescents in the ASD group were language delayed hence equivalent age was a more appropriate index compared with standard scores (Anderson et al., 2007; Elliott, 1990; Taylor et al., 1998) – and the level of narrative cohesion reached by the story in the Bears Family Projective Test (Table 1).

### Instruments

The ASD group was administered the Reynolds RIAS intelligence test (Reynolds & Kamphaus, 2003), the SENA Children and Adolescents Assessment System (Fernández-Pinto et al., 2015) and the Bears Family Projective Test (Bornstein & Tamis-lemonda, 1995; Esposito et al., 2018; Iandolo, 2011; Iandolo et al., 2012a, 2012b). The TD group was administered the Wechsler intelligence test: WPPSI-3 (Wechsler, 2002) and WISC-IV (Wechsler & Corral, 2007), the CBCL 4/18 multi–information questionnaire of Achenbach (Achenbach, 1991) and the Bears Family Projective Test. Both the RIAS and the Wechsler test provide comparable IQ scores. The CBCL 4/18 and SENA provide behavioural information in different contexts (school, family, self-perception) about competencies and eventual problematic behaviours. The RIAS intelligence test (Reynolds & Kamphaus, 2003) is an individually applied test from 3 years that provides a verbal intelligence index (IV), a non-verbal intelligence index (INV), a verbal memory index (IM) and a general IQ (IG). The child and adolescent evaluation system SENA (Fernández-Pinto et al., 2015) is a multi–information questionnaire focused on collecting behavioural, relational and functional information of the individual in different development contexts (parental, school and private information) from 3 years of age.

The Bears Family Projective Test (Bornstein & Tamis-lemonda, 1995; Esposito et al., 2018; Iandolo, 2011; Iandolo et al., 2012a, 2012b) is a thematic projective method of narrative test with a standard administration system that allows the stimulation and evaluation of narration from 3 years of age. The test involves providing the child (game format) or the adolescent (photographic format) with a set of small dolls and dramatic material from a family of anthropomorphic bears, for 10 minutes, to then tell a story in a time limit of 5 minutes. The test is videotaped and the narrated story is transcribed verbatim and analysed according to the Integrated System of Analysis of the Bears Family (Iandolo, 2011). The final indices of narrative analysis are divided into two report areas: formal and content aspects. The following formal aspects were evaluated: the number of propositions, episodes, the index of narrative cohesion and narrative structure. The following aspects of content were evaluated: the number of problematic events with and without solution, the use and location of the characters, the number of positive and negative relationships between characters, the number of adaptive and maladaptive behaviours set in motion by the characters of the story (see Supplemental material).

### Procedure

Children and adolescents with ASD were evaluated individually between 2016 and 2017 in Spain. The behavioural and operational data were derived from families, teachers and the participants themselves through the SENA questionnaire in previous sessions. In the first 45-minute individual session with each participant, the RIAS intelligence test was administered. In a second individual session, the Bears Family Projective Test was administered. Children and adolescents with TD were evaluated individually between 2008 and 2011 in Spain. The behavioural data were derived from the families through the CBCL questionnaire 4/18 in previous sessions. In the first 45-minute individual session, a first part of the Wechsler Intelligence test was administered. In a second 45-minute individual session, the second part of the Wechsler test and the Bears Family Projective Test were administered. For story analysis, two judges blinded to the research hypotheses were trained to code the videotaped sessions using the Bears Family Test Manual (Iandolo, 2011). The reliability was evaluated in 40% of the sample using Cohen’s kappa index, which was found to be statistically acceptable (kappa = 0.91). The results of the two groups were compared by matching them according to sex, socioeconomic level, equivalent age of the Total IQ, the level of narrative cohesion reached in the story of the Bears Family Projective Test (Table 1).

## Results

The two groups were first matched by equivalent age (Verbal, Total IQ), sex, socioeconomic level and narrative cohesion index. With respect to the Bears Family Projective Test story form, there were no significant differences between both groups in terms of the level of cohesion (*t* = 0.051, *p* = 0.96) and the narrative structure of the story (*t* = 0.096, *p* = 0.92) (Table 2). Results showed a significant difference between the ASD group and the TD group in chronological age (*t* = 2.10, *p* = 0.05) but not in equivalent age (*t* = 0.051, *p* = 0.96) (Table 3). Considering that some adolescents of the ASD group were language delayed, equivalent age was a more appropriate index compared with standard scores (Anderson et al., 2007; Elliott, 1990; Taylor et al., 1998).

To test Hypothesis 1, the correlations between narrative structure and cohesion with (i) chronological age and (ii) equivalent age were calculated. In both groups, there was a significant correlation between both chronological (ASD group; *r* = 0.50, *p* = 0.01, TD group; *r* = 0.55, *p* = 0.01) and equivalent age (ASD group; *r* = 0.54, *p* = 0.01, TD group; *r* = 0.55, *p* = 0.01) with the cohesion index, indicating a gradual progression of the cohesion index at a later age. However, significant correlations between both chronological (*r* = 0.48, *p* = 0.02) and equivalent age (*r* = 0.51, *p* = 0.01) with narrative structure was only found in the ASD group whereas those for the TD group were not significant (Table 4). These results mostly support Hypothesis 1, with the exception of the correlation between narrative structure and age in the TD group. Apart from this result, significant positive correlations were found between age (both chronological and equivalent age) and the structure and cohesion index, suggesting the improvement of the structure of the story with age.

For Hypothesis 2, t-tests were conducted to test for the differences in story features, in terms of propositions and episodes, between the ASD group and TD group. The stories narrated by the ASD group had a significantly higher number of propositions (*t* = −2.53, *p* = 0.05) and episodes (*t* = −3.28, *p* = 0.02) compared to the TD group (Table 5), supporting Hypothesis 2.

**Figure 1.**
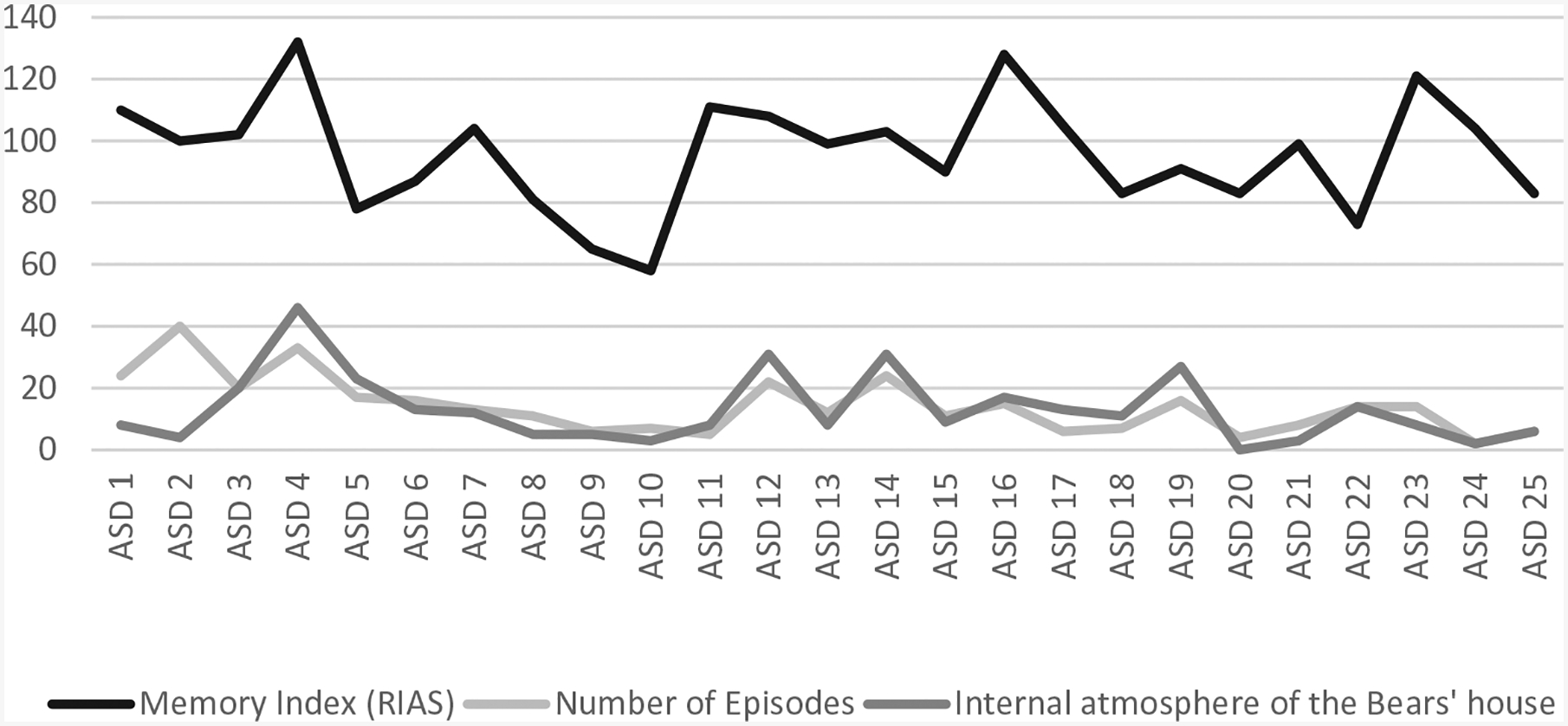
Adaptive and aggressive behaviours, solved problems, rule rejections and ambience (ASD & TD).

**Figure 2.**
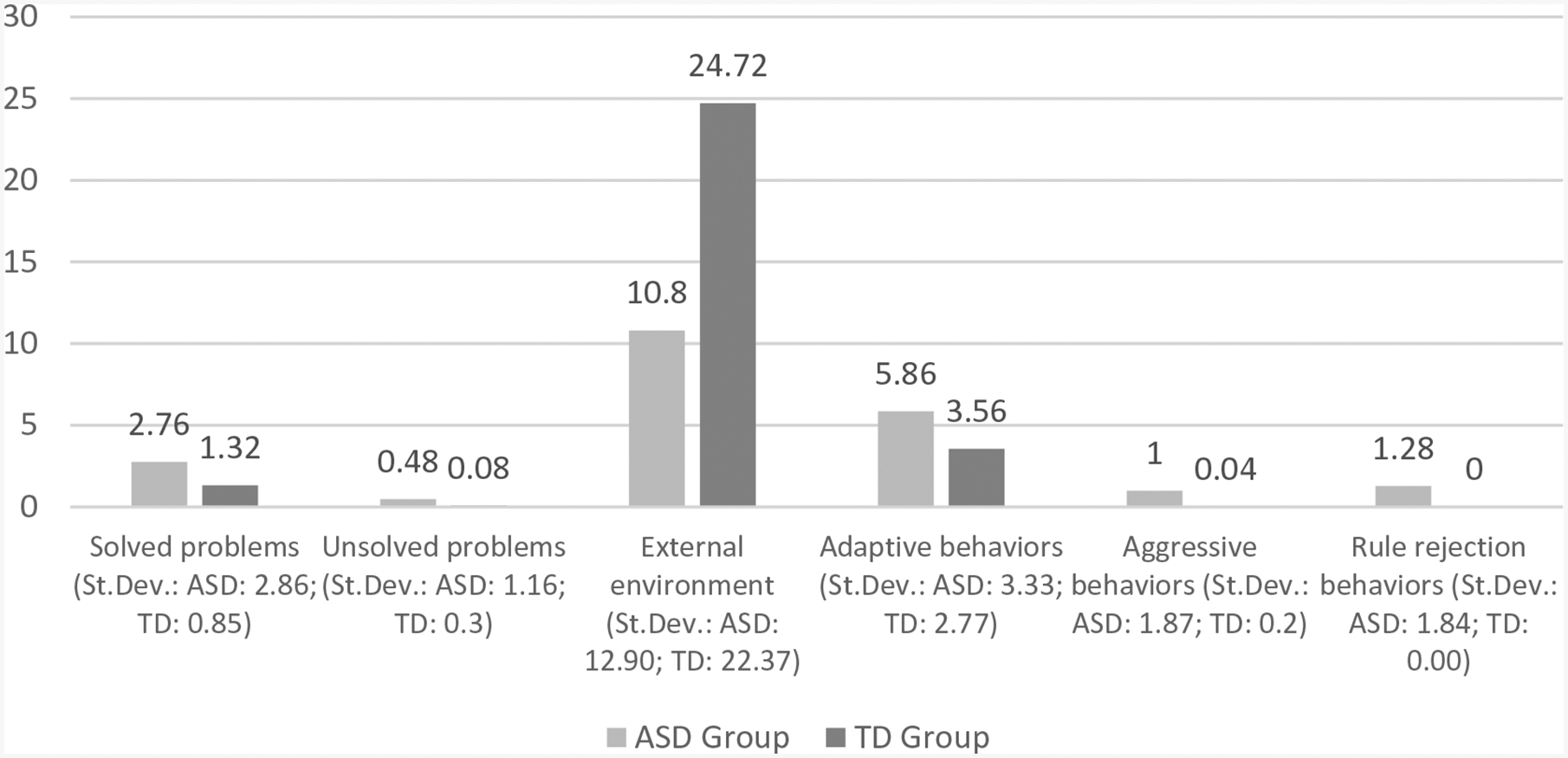
Age, adaptive behaviours and use of internal atmosphere of the Bears’ house (ASD).

For Hypothesis 3, t-tests were conducted to test for differences in story contents between the ASD group and TD group. With respect to the contents of the story, compared to the TD group, the results indicate that in the ASD group, there was greater use of adaptive behaviours (*t* = 2.45, *p* = 0.05), in addition to high variability (σ > μ) associated with a higher frequency of problems solved (*t* = 2.40, *p* = 0.05), aggressive behaviours (*t* = 2.55, *p* = 0.05), rule rejection behaviours (*t* = 2.55, *p* = 0.05) and less use of an external environment outside the house as the story’s setting (*t* = −2.69, *p* = 0.01) (Figure 1). Further, compared to the TD group, the stories narrated by the ASD group show a greater use of adaptive behaviours (*t* = 2.45, p = 0.05) and less use of an external environment outside the house as the story’s setting (*t* = −2.69, p = 0.01) (Figure 2).

Correlations between indices of story form and indices of story content were calculated, as well as the correlations between these indices and verbal, non-verbal and total IQ. The results indicate two different patterns in the stories of the two groups. Firstly, in the stories of the ASD group, the more propositions and episodes the story presents, the more adaptive behaviours, rejection of rules and use of the external environment outside the home as the story’s setting were recorded (Table 6). Secondly, in the stories of the TD group, the more episodes the story presents, the more adaptive behaviours, solved problems and use of the external environment outside the home as the story’s setting were recorded (Table 7). The number of propositions in the stories of the TD group was only significantly correlated with adaptive behaviours and solved problems (Table 7).

**Figure 3.**
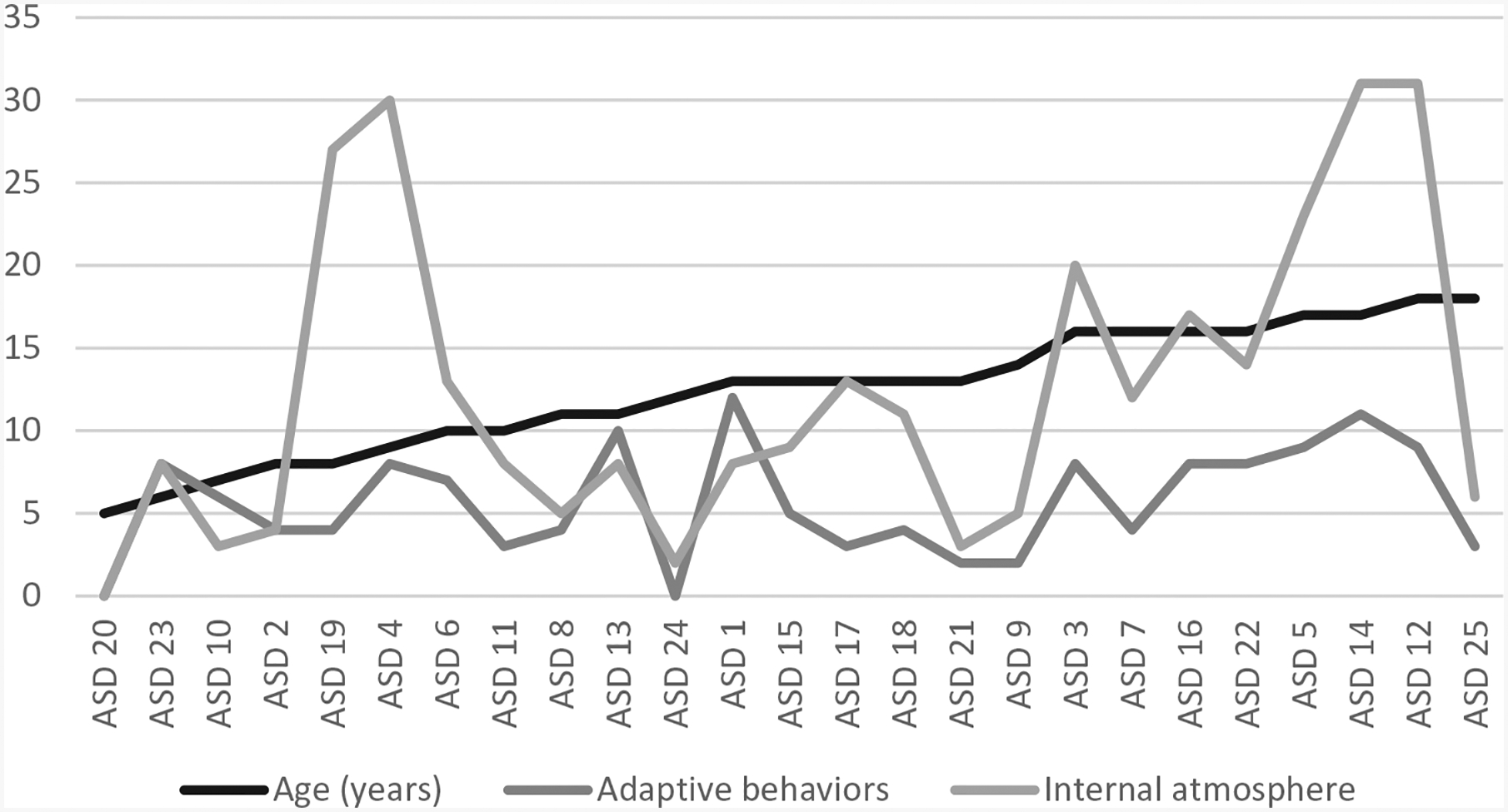
Verbal memory index (RIAS), number of episodes and frequency of interior ambience (ASD).

In addition, in the ASD group, the results indicate a positive correlation between the verbal memory index (RIAS), the number of episodes in the story (*r* = 0.41, *p* = 0.01) and the frequency of the house as the story’s setting (*r* = 0.42, *p* = 0.05). In other words, this means that a greater verbal memory competence corresponds to a story with a setting centred within the house itself. These correlations are not recorded in the TD group where, with higher verbal IQ (WISC-IV), there tend to be fewer unclear settings (*r* = −0.47, *p* = 0.05) and fewer non-clear characters (*r* = −0.51; *p* = 0.05) (Figure 3).

To test Hypothesis 4, the correlations between anxiety symptoms derived from the SENA questionnaire and story contents in the ASD group were calculated. When the story content elements that characterized the stories of the ASD group most were taken into consideration (adaptive behaviours, aggressive behaviours, high frequency of problems, and rejection of rules), it was found that they were significantly related to the intensity of anxious symptomatology as indicated by the participants themselves, the relatives and the school on the SENA questionnaire (Table 8).

## Discussion

No differences were expected between the two groups in the form of the story, due to the matching criteria. Although significant correlations between the cohesion index and the chronological and equivalent ages of both groups were found, the correlation between the narrative index and chronological and equivalent ages was only significant for the ASD group. Hence, the results only partially confirmed Hypothesis 1. These results differed from a previous study conducted in 4–10 years old children with typical development which found that cohesion and structure indexes followed linear trajectories of development (Esposito et al., 2018). A possible explanation for this difference could be the small sample size (TD group; *n* = 25) and inclusion of adolescents up to 18 years old in the present study. It may be that mastery of narrative structure peaks in late childhood for children with typical development, after which there is no significant difference in narrative structure with age.

The results indicate that despite there being no differences in cohesion and narrative structure, the stories of the ASD group tend to present higher propositions and episodes with more words relative to the TD group, confirming Hypothesis 2. It was found that the ASD group needed more propositions to describe an event, a tendency that usually manifests in younger children who, to express an idea, give more detours (Esposito et al., 2018; Iandolo, 2011; Iandolo et al., 2012b). Hence, this result corroborates with existing research

As in previous studies with the Bears Family Projective Test, the cohesion and narrative structure in both groups increase with age and according to language and vocabulary skills (Iandolo et al., 2012b). In addition, in the ASD group, there is a positive relationship between the verbal mnemonic competence and number of episodes of the story. Similar results were found in the study by Haebig et al. (2015), suggesting that the lexical-semantic knowledge of children with ASD may be immature, but with a similar organization to children with TD. As such, eventual differences between the stories of individuals with ASD and those with TD may be due to abilities related to expressive language. In other classic studies (see Botvin & Sutton-Smith, 1977; Bruner, 1991; Nelson, 1989; Stadler & Ward, 2005), it has been emphasized that development of narrative skills is gradual in childhood and youth stages. Around 7–8 years of age, the child develops a more advanced logical competence related to physical reality, allowing greater competence in solving problems and logical-narrative coherence (Esposito et al., 2018; Iandolo et al., 2012b).

Differences in story contents were expected between the two groups due to differences in relational difficulty between the two groups that could have been reflected in the story. The results indicate that the stories of the ASD group present more adaptive behaviours of the characters and, taking into account a high variability among subjects, more frequent problems, aggressive behaviours, rejection of rules and less use of the external environment outside the home as the story’s setting, which confirmed Hypothesis 3. The results indicated that these contents that most characterize the ASD group were related to the intensity of the anxious symptomatology indicated by the participants themselves, the family members and the school, confirming Hypothesis 4 as well. In previous research with children with typical development, after 6 years of age, children tend to set the story less inside the house, probably because with the start of primary school, children are more open to the outside world (Iandolo, 2011). In addition, in children without behavioural difficulties, aggressive and adaptive behaviours are not detected at the same level as in the ASD group (Iandolo et al., 2012b). The results also indicate that the contents of the story of the Bears Family are related to experiences, behaviours and symptoms of anxiety in different contexts (self, family, school). As indicated in the study by Losh and Capps (2003), the introduction of personal experiences in stories of children with ASD is often done in more indirect terms (problems, adaptation, experiences at home), where verbal memory plays an important role (Diehl et al., 2006). This could be a possible explanation for the frequent setting of the story of the ASD group within the house, unlike the TD group. Furthermore, the contents of the ASD group tend to be less balanced between adaptive and maladaptive, positive and negative behaviours, probably reflecting less integration of cognitive, emotional and social components (Happé & Frith, 2006; Plaisted, 2001).

This is one of the first studies to examine the relationship between story contents of children with ASD and anxious symptomatology as indicated by themselves and other contexts including their parents and schools. While a previous study has found the relation of children’s narratives with predicting later childhood anxiety, results from the present study suggest that the internal representations of children with ASD can be observed from key characteristics of the contents of their story. Hence, narratives can be used to examine these representations and serve as a possible avenue to identify children with ASD who may be experiencing anxiety. Findings in this study also highlight the possible use of narrative therapy as a means of externalization for children with ASD, given that certain story features of narratives of children with ASD have been found to be related to anxious symptomatology. The narrative therapy technique of externalization (White, 2007) can help children with ASD to externalize their emotions and develop self-regulation skills. It can be used to engage children with autism to define concrete problems and exploring plausible courses of action and solutions, through which they can learn to solve future problems while serving to reduce their distress. A number of studies have indicated that narrative therapy has been helpful in working with individuals with learning disabilities (Lambie & Milsom, 2010), communication difficulties (Wolter et al., 2006) and ASD (Cashin et al., 2012).

## Conclusion

The results show no differences in the formal quality of the story in both groups of the study but variations in the formal quantity with more episodes and propositions used by the ASD group. Although the lexical-semantic knowledge of the ASD group may be more immature, it shares a similar organization to children with TD. This can be observed from the parallelism in the responses provided by individuals with TD and ASD when describing a situation of discomfort (Ayuda-Pascual & Martos-Pérez, 2007), but children with ASD tend to avoid the exposure of situations with a strong emotional basis rather than ‘disguising’ the situation, as do children with TD. The narrations of both related groups are associated with their personal experiences and emotional-behavioural aspects, but children and adolescents with ASD express this association in a more indirect way. In the ASD group, there is greater use of maladaptive behaviours, more problems and rejection of rules in their stories and greater traits of anxiety, rigidity and isolation being recognized in both family and school contexts. The correlation between these story contents and anxious symptomatology as identified by the self and others found for children with ASD suggests that their narratives can be important sources of information in terms of identifying the emotional and behavioural problems as well as anxiety experienced by these individuals.

## Supplementary Material

Supplementary Material

## Figures and Tables

**Table 1.** Characteristics of the sample: ASD Group (n = 25, Males); TD Group (n = 25, Males).

		Min	Max	Average	SD	t
Chronological age - years	ASD - Group	5	18	12,40	3,83	2,1 (p 0,05)
	TD - Group	5	17	10,22	3,52	
Equivalent age (IQ) - years	ASD - Group	5	17	11,56	3,61	1,319 (p 0,20)
	TD - Group	5	17	10,22	3,52	
Verbal IQ *	ASD - Group (RIAS)	55	125	89,96	18,42	t - 3,022 (p 0,01)
	TD - Group (Wechsler)	90	120	102,76	9,7	
Non-Verbal IQ *	ASD - Group (RIAS)	53	136	97,68	18,8	t - 1,20 (p 0,23)
	TD - Group (Wechsler)	89	122	105,04	8,91	
Total IQ *	ASD - Group (RIAS)	56	134	93	17,97	t - 3,002 (p 0,01)
	TD - Group (Wechsler)	91	128	102,08	7,92	
Socioeconomic Level **	ASD - Group	12	55,5	29,3	12,21	t - 0,17 (p 0,86)
	TD - Group	12	52	29,84	9,986	
Cohesion Index (Story) ***	ASD - Group	1	11	7,72	2,685	t 0,051 (p 0,96)
	TD - Group	1	11	7,68	2,883	
Internalization- Family (CBCL/4–18)^[]^	TD - Group	41	52	43,30	5,20	
Externalization - Family (CBCL/4–18)^[]^	TD - Group	37	57	49,32	8,63	//
Internalization- School (TRF/5–18)^[]^	TD - Group	44	55	48,43	6,11	
Externalization - School (TRF/5–18)^[]^	TD - Group	42	53	47,32	7,35	
Global Problem Index - Family (SENA)^[]^	ASD - Group	42	101	66,33	12,9	
Emotional problems Index - Family (SENA)^[]^	ASD - Group	41	94	64,19	13,70	
Behavioural problems Index - Family (SENA)^[]^	ASD - Group	41	99	62,38	17,30	
Exec. Functions Probl. Index - Family (SENA) ^[]^	ASD - Group	52	88	68,24	11,62	//
Personal Resources Index - Family (SENA)^[]^	ASD - Group	10	45	25,71	10,50	
Global Problem Index - School (SENA)^[]^	ASD - Group	44	85	60,08	12,71	
Emotional problems Index - School (SENA)^[]^	ASD - Group	46	66	56,46	5,65	
Behavioural problems Index - School (SENA)^[]^	ASD - Group	43	111	57,46	21,18	
Exec. Functions Probl. Index - School (SENA)^[]^	ASD - Group	50	86	67,31	12,46	
Personal Resources Index - School (SENA)^[]^	ASD - Group	22	48	35,38	8,71	

* IQ scores: mean of 100 and standard deviation of 15.

** Socio-Economic Status (Hollingshead): 10–19,5 low; 20–29,5 medium-low; 30–39,5 medium; 40–54,5 medium-high; > 55 high.

*** Bears Family Cohesion Index: scalar score 1–11.

[] T-scores: mean of 50 and standard deviation of 10.

// The t-test is not available because in the two groups two different questionnaires have been used (SENA for ASD group and CBCL-ASEBA for TD-group)

**Table 2.** Structure and cohesion index differences between groups (ASD & TD).

		Min	Max	Average	SD	t
Structure Index (Story Form) *	ASD - Group	1	6	3,72	1,54	t 0,096 (p 0,92)
	TD - Group	1	6	3,68	1,41	
Cohesion Index (Story Form) **	ASD - Group	1	11	7,72	2,685	t 0,051 (p 0,96)
	TD - Group	1	11	7,68	2,883	

** Bears Family Structure Index: scalar score 1–6.

** Bears Family Cohesion Index: scalar score 1–11.

**Table 3.** Chronological and equivalent age differences between groups (ASD & TD).

		Min	Max	Average	SD	t
Chronological age - years	ASD - Group	5	18	12,4	3,83	**t 2,10 (p 0,05)**
	TD - Group	5	17	10,22	3,52	
Equivalent age (IQ) - years	ASD - Group	5	17	11,56	3,61	t 0,051 (p 0,96)
	TD - Group	5	17	10,22	3,52	
Verbal IQ *	ASD - Group (RIAS)	55	125	89,96	18,42	**t - 3,022 (p 0,01)**
	TD - Group (Wechsler)	90	120	102,76	9,7	
Non-Verbal IQ *	ASD - Group (RIAS)	53	136	97,68	18,8	t - 1,20 (p 0,23)
	TD - Group (Wechsler)	89	122	105,04	8,91	
Total IQ *	ASD - Group (RIAS)	56	134	93	17,97	**t - 3,002 (p 0,01)**

**Table 4.** Correlations of story features with chronological and equivalent age (ASD & TD).

			Chronological Age	Equivalent Age
Bears Family - Story Form	**Number of propositions**	ASD - Group	r 0,24 p 0,23	r 0,28 p 0,18
		TD - Group	**r 0,90 p 0,01**	**r 0,90 p 0,01**
	**Number of episodes**	ASD - Group	r 0,01 p 0,94	r 0,034 p 0,82
		TD - Group	**r 0,56 p 0,01**	**r 0,56 p 0,01**
	**Cohesion index**	ASD - Group	**r 0,50 p 0,01**	**r 0,54 p 0,01**
		TD - Group	**r 0,55 p 0,01**	**r 0,55 p 0,01**
	**Structure index**	ASD - Group	**r 0,48 p 0,02**	**r 0,51 p 0,01**
		TD - Group	r 0,28 p 0,16	r 0,28 p 0,16
Bears Family - Story Content	**Adaptive behaviours**	ASD - Group	r 0,29 p 0,16	r 0,34 p 0,09
		TD - Group	r 0,26 p 0,21	r 0,26 p 0,21
	**Solved problems**	ASD - Group	r 0,38 p 0,06	r - 0,38 p 0,06
		TD - Group	**r 0,41 p 0,05**	**r 0,41 p 0,05**
	**Rule rejection behaviours**	ASD - Group	r - 0,52 p 0,80	r - 0,20 p 0,92
		TD - Group	//	//
	**External atmosphere**	ASD - Group	r - 0,17 p 0,41	r - 0,13 p 0,53
		TD - Group	r 0,27 p 0,19	r 0,27 p 0,19

**Table 5.** Story features differences between groups (ASD & TD).

						Univariate General Lineal Model (Equivalent Age as fixed factor)	
			Average	SD	t	F	Partial eta squared
Bears Family - Story Form	**Number of propositions**	ASD – Group TD - Group	49 31	28 22	t - 2,53 (p 0,05)	F (18,31) = 2,83, p.005	0,622
	**Number of episodes**	ASD – Group TD - Group	25 14	14 9	t - 3,28 (p 0,01)	F (18,31) = 0,07, p.067	0,516
	**Cohesion index**	ASD – Group TD - Group	7,68 7,72	2,88 2,69	t 0,05 (p 0,96)	F (18,31) = 5,85 p.001	0,773
	**Structure index**	ASD – Group TD - Group	3,68 3,72	1,41 1,54	t 0,09 (p 0,92)	F (18,31) = 2,28 p.021	0,570
Bears Family - Story Content	**Adaptive behaviours**	ASD – Group TD - Group	5,68 3,56	3,33 2,77	t 2,45 (p 0,05)	F (12,12) = 1,91 p.137	0,657
	**Solved problems**	ASD – Group TD - Group	2,76 1,32	2,86 0,85	t 2,40 (p 0,05)	F (12,12) = 2,35 p.076	0,702
	**Rule rejection behaviours**	ASD – Group TD - Group	1,28 0	1,84 0	t 2,55 (p 0,05)	//	//
	**External atmosphere**	ASD – Group TD - Group	10,80 24,72	12,90 22,36	t - 2,69 (p 0,01)	F (12,12) = 1,44 p.267	0,591

The statistical index is not available because, in the stories of the TD group, no rules rejection behaviours are registered.

**Table 6.** ASD within group correlations.

		Age & IQ					Story Form				Story Content			
ASD within group correlations (n = 25)		Chron. Age	Equiv. Age	Verbal IQ	N-Verb. IQ	Total IQ	N. Prop.	N. Epis.	Cohesion	Structure	Adaptive	Solved prob.	Rule reject.	Extern. atmos.
Age & IQ	Chron. Age	**1**	**r 0.95, p 0.01**	r - 0.22, p 0.28	r - 0.10, p 0.96	r - 0.13, p 0.52	r 0.24, p 0.24	r 0.01, p 0.94	**r 0.50, p 0.01**	**r 0.48, p 0.01**	r 0.29, p 0.16	r 0.38,p 0.06	r - 0.05, p 0.80	r - 0.17, p 0.41
	Equiv. Age		**1**	r - 0.30, p 0.14	r 0.02, p 0.91	r - 0.15, p 0.46	r 0.27, p 0.18	r 0.03, p 0.87	**r 0.54, p 0.01**	**r 0.51, p 0.01**	r 0.34, p 0.09	r 0.38, p 0.06	r - 0.02, p 0.92	r - 0.13, p 0.54
	Verbal IQ			**1**	**r 0.46, p 0.05**	**r 0.83, p 0.01**	r 0.13, p 0.54	r 0.13, p 0.53	r - 0.11, p 0.61	r - 0.09, p 0.65	r 0.01, p 0.95	r - 0.09, p 0.66	r - 0.08, p 0.72	r 0.01, p 0.93
	Non-Verbal IQ				**1**	**r 0.87, p 0.01**	r 0.20, p 0.34	r 0.14, p 0.50	r 0.32, p 0.12	r 0.08, p 0.68	r 0.26, p 0.21	r 0.15, p 0.47	r- 0.11, p 0.57	r - 0.12, p 0.95
	Total IQ					**1**	r 0.20, p 0.34	r 0.16, p 0.44	r 0.15, p 0.47	r 0.02, p 0.91	r 0.17, p 0.40	r 0.04, p 0.84	r- 0.13, p 0.54	r 0.00, p 0.95
Story Form	Numb. propositions						**1**	**r 0.90, p 0.01**	**r 0.42, p 0.05**	r 0.21, p 0.30	**r 0.73, p 0.01**	r 0.38, p 0.60	**r 0.58, p 0.01**	**r 0.61, p 0.01**
	Numb, episodes							**1**	r 0.26, p 0.20	r 0.09, p 0.67	**r 0.57, p 0.01**	r 0.34, p 0.09	**r 0.77, p 0.01**	**r 0.77, p 0.01**
	Cohesion index								**1**	**r 0.52, p 0.01**	**r 0.71, p 0.01**	**r 0.53, p 0.01**	r - 0.10, p 0.63	r - 0.05, p 0.80
	Structure index									**1**	r 0.23, p 0.26	r 0.35, p 0.08	r- 0.10, p 0.62	r - 0.14, p 0.48
Story Content	Adaptive behaviours										**1**	**r 0.1158, p 0.01**	r 0.13, p 0.53	r 0.20, p 0.34
	Solved problems											**1**	r 0.29, p 0.16	r 0.00, p 0.99
	Rule rejection												**1**	**r 0.75, p 0.01**
	External atmosphere													**1**

**Table 7.** TD within group correlations.

		Age & IQ					Story Form				Story Content			
TD within group correlations (n = 25)		Chron. Age	Equiv. Age	Verbal IQ	N-Verb. IQ	Total IQ	N. Prop.	N. Epis.	Cohesion	Structure	Adaptive	Solved prob.	Rule reject.	Extern. atmos.
Age & IQ	Chron. Age	**1**	**r 0.99, p 0.01**	r - 0.39, p 0.06	**r - 0.70, p 0.01**	**r - 0.62, p 0.01**	**r 0.90, p 0.01**	**r 0.56, p 0.01**	**r 0.56, p 0.01**	**r 0.28, p 0.01**	r 0.26, p 0.21	**r 0.41, p 0.01**	//	r 0.26, p 0.19
	Equiv. Age		**1**	r - 0.39, p 0.06	**r - 0.70, p 0.01**	**r - 0.62, p 0.01**	**r 0.90, p 0.01**	**r 0.56, p 0.01**	**r 0.56, p 0.01**	**r 0.28, p 0.01**	r 0.26, p 0.21	**r 0.41, p 0.01**	//	r 0.26, p 0.19
	Verbal IQ			**1**	**r 0.42, p 0.05**	**r 0.64, p 0.01**	r - 0.31, p 0.13	r 0.03, p 0.87	r 0.17, p 0.42	r 0.30, p 0.14	r 0.05, p 0.80	r 0.00, p 0.97	//	r 0.24, p 0.25
	Non-Verbal IQ				**1**	**r 0.87, p 0.01**	**r - 0.77, p 0.01**	**r - 0.53, p 0.01**	**r - 0.43, p 0.05**	r - 0.29, p 0.15	r - 0.38, p 0.60	**r - 0.50, p 0.01**	//	r - 0.16, p 0.45
	Total IQ					**1**	**r - 0.62, p 0.01**	r - 0.21, p 0.31	r - 0.28, p 0.18	r - 0.02, p 0.89	r - 0.05, p 0.79	r - 0.22, p 0.28	//	r 0.12, p 0.58
Story Form	Numb. propositions						**1**	**r 0.78, p 0.01**	**r 0.58, p 0.01**	r 0.37, p 0.07	**r 0.43, p 0.05**	**r 0.56, p 0.01**	//	r 0.32, p 0.10
	Numb, episodes							**1**	**r 0.61, p 0.01**	**r 0.54, p 0.01**	**r 0.76, p 0.01**	**r 0.77, p 0.01**	//	**r 0.71, p 0.01**
	Cohesion index								**1**	**r 0.74, p 0.01**	**r 0.60, p 0.01**	**r 0.74, p 0.01**	//	**r 0.47, p 0.05**
	Structure index									**1**	**r 0.56, p 0.01**	**r 0.71, p 0.01**	//	r 0.35, p 0.08
Story Content	Adaptive behaviours										**1**	**r 0.84, p 0.01**	//	**r 0.57, p 0.01**
	Solved problems											**1**	//	**r 0.57, p 0.05**
	Rule rejection												**1**	//
	External atmosphere													**1**

**Table 8.** Correlations between anxiety symptoms (SENA questionnaire) and contents of story (ASD).

		Bears Family Story			
		Solved problems	Adaptive behaviours	Aggressive behaviours	Rule-rejecting behaviours
SENA questionnaire	Anxiety (Self-report)		r 0.40, p 0.01, N22		
	Anxiety (Family)		r 0.46, p 0.05, N21		
	Anxiety (School)	r 0.48, p 0.05, N13			
	Emotional Intelligence (School)			r - 0.40, p 0.05, N21	
	Depression (School)				r 0.48, p 0.05, N13
	Disposition to studying (School)				r - 0.50, p 0.05, N12

Categories of analysis of the story of the Bears Family Narrative Test
