# Supplementary figures and images for: Story contents and intensity of the anxious symptomatology in children and adolescents with Autism Spectrum Disorder
Source: Int J Adolesc Youth. Author manuscript; Available in PMC 2020 Apr 13. (PMC7153759; doi:10.1080/02673843.2020.1737156)

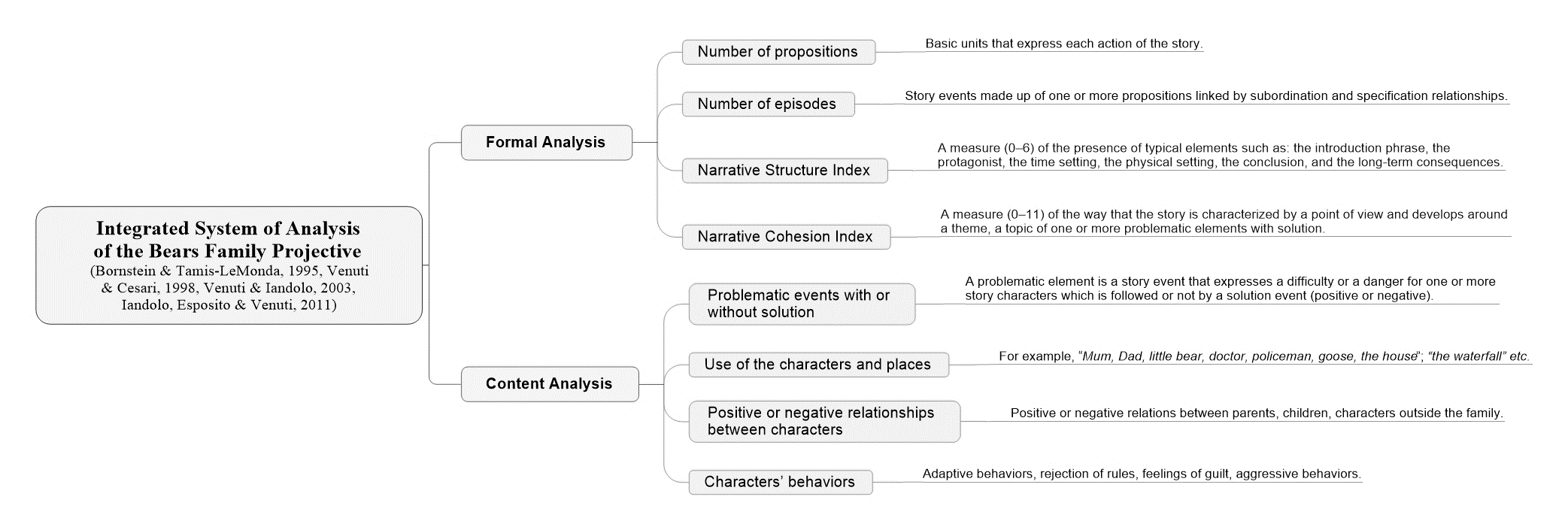


Categories of analysis of the story of the Bears Family Narrative Test

Supplement: Supplementary Material [file NIHMS1571054-supplement-Supplementary_Material.docx]
